# Red ear syndrome: a case series and review of the literature

**DOI:** 10.1186/s13256-024-04485-4

**Published:** 2024-07-09

**Authors:** Grace M. Callan, Frederick Freitag, Amir Soheil Tolebeyan

**Affiliations:** 1 Medical College of Wisconsin, Milwaukee, USA; 2 Tufts Medical Center, Tufts University School of Medicine, Boston, USA

**Keywords:** Red ear syndrome, Migraine, Aura, Erythromelalgia

## Abstract

**Background:**

Red Ear Syndrome is a burning sensation and erythema of the ear, associated with a various number of disorders including migraine, trigeminal neuralgia, autoimmune disorders etc. Theories for RES pathophysiology have developed from current understandings of comorbid conditions. Characterizing the underlying mechanism of RES is crucial for defining effective treatments.

**Case presentation:**

Three caucasian patients, ages 15, 47, and 67 years, with migraine, one with erythromelalgia are reported in this manuscript. RES pathophysiology is not fully understood due to its variable clinical presentation and numerous comorbid conditions, making it difficult to identify effective treatments.

**Conclusion:**

RES seems to be largely treatment-resistant, and most options involve treating the associated disorders and minimizing pain. Further investigation of future cases should lead to a more comprehensive understanding of the fundamental cause of RES and, hopefully, successful treatments.

## Introduction

Red Ear Syndrome (RES), described in 1996 by Lance, is a rare phenomenon characterized by burning sensations and erythema of the external ear [1]. The causes of this clinical presentation are not understood, although RES has generally been linked to migraine, trigeminal autonomic cephalalgia (TAC), erythromelalgia, vasculitis, and systemic lupus erythematosus (SLE). The clinical presentation of RES is quite variable. Pediatric, adolescent, and adult cases have been reported. Duration ranges from a few minutes to several hours. The redness can be bilateral or unilateral and may extend to the skin around the ear. Attacks may be spontaneous or triggered by touch and temperature changes.

In an attempt to further describe its presentation, RES is now divided into primary (or idiopathic) and secondary forms. Primary RES is associated with chronic migraine, more frequently seen in the pediatric and adolescent populations [2, 3]. RES in children is most commonly seen associated with migraine as opposed to other types of primary headache; furthermore, in these cases, it seems that RES occurs bilaterally, sometimes during the migraine attack, as well as concurrently with phonophobia, vomiting, and throbbing pain, especially in males [4]. On the other hand, secondary RES occurs mainly in adults who have upper cervical spine disorders, trigeminal neuralgia, thalamic syndrome, or temporomandibular joint dysfunction [2, 3]. While RES with migraine is more often seen in children, adults can also present with both. A study which looked at adult patients who had migraine (with or without aura) and/or temporomandibular joint dysfunction found that RES attacks did not always occur at the same time as their migraines, were unilateral, and often triggered by temperature changes; moreover, some adults had lower facial cluster headaches or autonomic symptoms [5]. At this time, most treatments for RES include prophylactic and abortive treatments for migraine, which have variable effects in mitigating symptoms.

Additional cases have been reported in order to characterize RES; however, its etiology, pathophysiology, and treatments continue to be ambiguous. As a result, most clinicians reach a diagnosis by exclusion and are challenged to find the appropriate treatment for patients. In this review, we are presenting three cases of migraine and RES, one pediatric and two adult cases, one of which also presents with erythromelalgia.

## Methodology

For this literature review, we identified sources in PubMed without date or language restrictions using keywords such as red ear syndrome, migraine, and erythromelalgia. Studies that did not discuss these topics were excluded. The search was completed by one reviewer. The process was supervised by two preceptors. Papers that included background information about RES, proposals for pathophysiology, case reports, and treatment options were reviewed.

### Case series

**Fig. 1:**
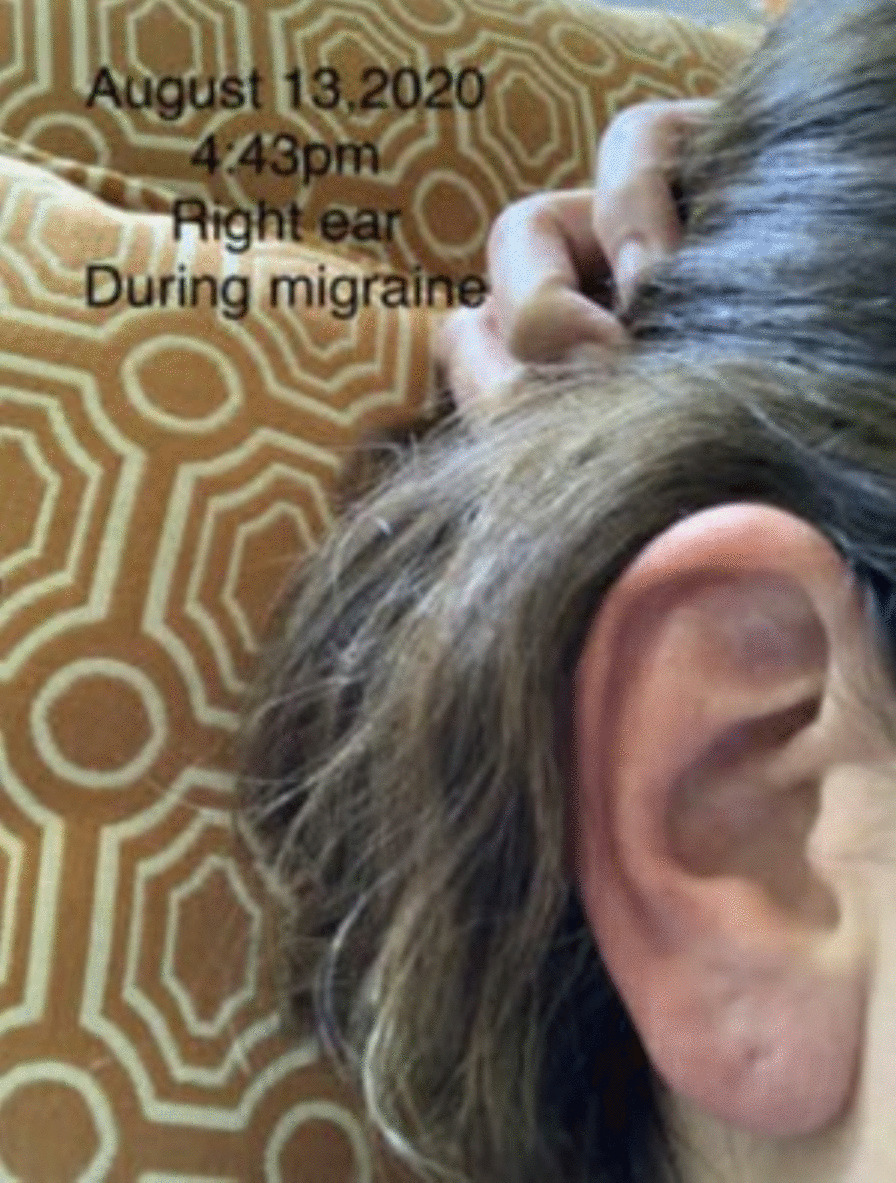
Right ear redness during a migraine attack

**Fig. 2:**
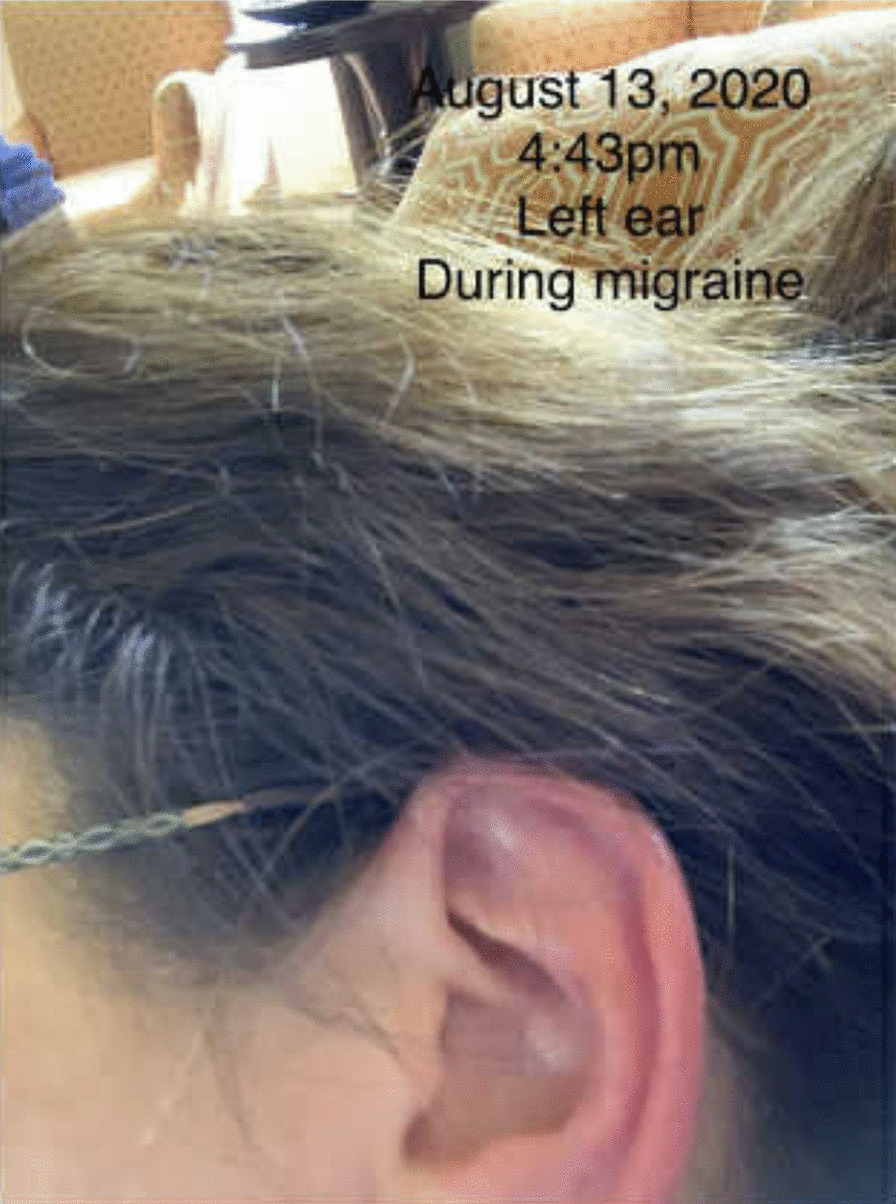
Left ear redness during a migraine attack

1. A 67-year-old caucasian female with chronic migraine aura presented with unilateral RES several times a week for about two hours. Her past medical history includes occipital neuralgia, rheumatoid arthritis, Raynaud’s disease, probable Ehlers-Danlos Syndrome, nasal septal ulcer, basal cell carcinoma of the face, and gastroesophageal reflux disease. Multiple migraine prophylactic agents failed. Finally, her headaches showed significant improvement after the initiation of Onabotulinum Toxin A, with attacks occurring once a week for half an hour [6].
2. A 47-year-old caucasian woman with a past medical history of erythromelalgia, Raynaud’s Syndrome, and episodic migraine without aura experienced intermittent reddening of either ear lasting about 10 min (see Figs. 1 and 2).

Her migraine symptoms include a constant pain emanating from her forehead, which sometimes can affect her neck and occipital area, typically beginning in the morning and lasting 3-7 hours, along with photophobia, phonophobia, and nausea. She also reports pain and a sensation of fullness in her ears. She can experience ptosis on the right side. She developed floaters in both eyes, and her eye exam indicated it was due to peripheral vascular disease. Her migraine was treated with nortriptyline, topiramate, and sumatriptan, although she stopped them due to side effects. She also used to take naproxen. She decided to take magnesium oxide for prophylaxis and ubrogepant for abortive therapy, which works well.

Her erythromelalgia is characterized by painful burning sensations in her hands and feet, although it is most pronounced in her feet. Episodes can wake her up in the middle of the night.

We think her symptoms may be related to an underlying autoimmune disease, although her testing has been unremarkable to this point. She was not interested in adding a medication such as valproic acid or venlafaxine to possibly treat both her erythromelalgia and migraine.

3. A 15-year-old caucasian girl with a past medical history of migraine with aura experiences sporadic ear redness and pain episodes. These episodes did not occur at the same time as her migraines, which are responsive to sumatriptan and ibuprofen.

## Results

Overall, RES is largely resistant to treatment. Most treatments are used regularly for primary headaches and have variable benefits. Such medications include NSAIDs (Indomethacin), Amitriptyline, Verapamil, Propranolol, and Gabapentin [2]. Botulinum toxin and cervical nerve blocks have also been utilized [2, 6, 7]. For one adult patient with vestibular migraine, relief of symptoms was achieved upon treatment with amitriptyline and valproic acid [8]. Amitriptyline also worked for a pediatric patient with a history of migraine without aura [9]. Verapamil and gabapentin showed improvement in adult patients with cluster headaches or autonomic dysfunction [5]. Ibuprofen showed improvement in adult patients with idiopathic RES [7]. Patients should also be encouraged to keep a symptom diary to identify any possible triggers, including diet or stress. Interestingly, one patient’s diary indicated that orange juice correlated with attacks, and avoiding this food helped reduce symptoms [10]. Based on our literature review, there are no successful non-pharmacologic treatments described for RES. As more cases are reported, a definitive understanding of the pathology will guide additional treatment options.

## Discussion

A differential diagnosis for an erythematous ear should be broad: trauma, temperature sensitivity, connective tissue disorder, and autoimmune disorder or vasculitis. The question remains if a red ear is a common symptom among several known pathologies, such as primary headache disorder and erythromelalgia, or if it is an entirely independent syndrome. Understanding the pathology of associated conditions as well as the innervation and vasculature of the ear has shed some light on possible underlying mechanisms of RES. Branches from nerve roots C2 and C3 innervate the earlobe, and the auriculotemporal nerve from the mandibular division of the trigeminal nerve innervates the anterosuperior ear and temporomandibular joint; the trigeminal nerve also innervates the vasculature supplying the ear which consists of the middle temporal and posterior auricular arteries, branches of the external carotid artery. A commonly proposed general mechanism for RES involves sympathetic inhibition and parasympathetic overactivation resulting in vasodilation. Nerve impulses lead to a release in vasodilator substances such as calcitonin gene-related peptide (CGRP) and substance P, as well as nitric oxide, resulting in vasodilation and subsequent pain [2, 11]. The accompanying symptoms of RES depend on which nerves send the initial impulses.

Concomitant conditions have led to a few more specific proposals for the mechanism behind RES. RES is most associated with primary headache disorders, such as migraine, cluster headache, and TACs. Of note, primary headaches can be accompanied by spontaneous extracranial hemorrhagic phenomena (SEHP), which are described as epistaxis, ecchymosis, and hematohidrosis [12]. Perhaps RES may similarly result in minor bleeding in patients with a history of a headache disorder. Previously, the release of vasodilator substances as a result of trigeminovascular activation during migraine attacks has been postulated as a potential cause of RES [2, 13]. Additionally, TAC is a combination of primary headache and autonomic symptoms such as lacrimation, rhinorrhea, and miosis; as a result, it has been hypothesized that RES may stem from a dysregulated brainstem trigemino-autonomic circuit with trigemino-parasympathetic activation [2]. Moreover, some patients’ symptoms were relieved by a C3 local anesthetic nerve block; it could be reasoned that Angry Back-firing C-nociceptors syndrome, due to a lower pain threshold triggered by mechanical stimulation, may be the cause of RES [2]. A more recent proposal combines several theories, proposing that RES is a result of central and peripheral dysfunction [14].

RES has also been connected with erythromelalgia, which is characterized by similar burning sensations and redness of the hands and feet. It has been proposed that erythromelalgia could be caused by vascular maldistributions, leading to hypoxia-induced neuropathy and arteriovenous shunting. Therefore, some researchers suggest that RES might be an auricular subtype [2, 15]. Primary erythromelalgia is a result of an autosomal dominant mutation in the SCN9A voltage-gated sodium channel on nociceptive neurons, primarily seen in younger patients [16]. While biopsies are rarely done for diagnosis, it has been seen that there is reduced nerve density in affected areas [16]. Medications targeting sodium channels, such as mexiletine and ranolazine, are typically used in these cases [16]. Secondary erythromelalgia may be a result of intimal and smooth muscle proliferation as well as thrombotic occlusions, and it has been associated with hematologic and connective tissue disorders, including but not limited to vasculitis, SLE, rheumatoid arthritis, and Sjögren Syndrome [16]. The prognosis of RES is variable and most likely dependent on control of concomitant medical issues, especially when it is associated with primary headaches. As more cases and beneficial treatments are reported, a definitive pathological understanding of RES will develop and, in turn, guide routine clinical protocol, especially when RES occurs with comorbid conditions.

## Conclusion

Although RES presents simply as a painful burning sensation and redness of the external ear, its pathophysiology and treatment have not been fully characterized owing to its variable clinical presentations. While RES is commonly reported with coexisting migraine, perhaps the most striking similarities may be seen with erythromelalgia. However, there are several associated conditions that present with this syndrome, making it difficult to completely describe its etiology. A comprehensive understanding of its fundamental cause and successful treatment regimens will need further investigation as future cases arise.

## Data Availability

The original contributions presented in the study are included in the article/supplementary material, further inquiries can be directed to the corresponding author.
